# Engineering a Highly Active Sucrose Isomerase for Enhanced Product Specificity by Using a “Battleship” Strategy

**DOI:** 10.1002/cbic.202000007

**Published:** 2020-04-16

**Authors:** Patrick Pilak, André Schiefner, Judith Seiboth, Johannes Oehrlein, Arne Skerra

**Affiliations:** ^1^ Lehrstuhl für Biologische Chemie Technische Universität München Emil-Erlenmeyer-Forum 5 85354 Freising Germany; ^2^ Evonik Creavis GmbH Paul-Baumann-Strasse 1 45772 Marl Germany

**Keywords:** enzyme engineering, isomaltulose, isomerases, protein design, sucrose, trehalulose

## Abstract

The sucrose isomerase SmuA from *Serratia plymuthica* efficiently catalyses the isomerisation of sucrose into isomaltulose, an artificial sweetener used in the food industry. However, the formation of a hygroscopic by‐product, trehalulose, necessitates additional separation to obtain a crystalline product. Therefore, we have improved the product specificity of SmuA by first introducing a few exploratory amino acid exchanges around the active site and investigating their influence. Then, we devised a second set of mutations, either at promising positions from the preceding cycle, but with a different side chain, or at alternative positions in the vicinity. After seven iterative cycles involving just 55 point mutations, we obtained the triple mutant Y219L/D398G/V465E which showed 2.3 times less trehalulose production but still had high catalytic efficiency (*k*_cat_/*K*_M_=11.8 mM^−1^ s^−1^). Not only does this mutant SmuA appear attractive as an industrial biocatalyst, but our semirational protein‐engineering strategy, which resembles the battleship board game, should be of interest for other challenging enzyme optimization endeavours.

## Graphical abstract

**A sweet victory**: A semirational protein engineering strategy inspired by the “battleship” board game was applied to systematically improve the product spectrum of the sucrose isomerase SmuA from *S. plymuthica* with regard to yielding the artificial sweetener isomaltulose at higher purity (94.7 %) and with significantly lower contamination of the hygroscopic by‐product trehalulose (1.5 %).

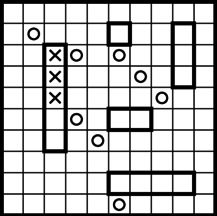

## Introduction

The sucrose isomer isomaltulose (6‐*O*‐α‐d‐glucopyranosyl‐d‐fructose, also referred to as palatinose) and the diastereomeric mixture of the corresponding sugar alcohols derived by catalytic hydrogenation, commonly known as isomalt,^1^ have attracted significant interest as safe low‐calorie sweeteners in the food industry. Replacement of sucrose by such compounds in food products or sweets both decreases the risk of bacterial tooth decay and attenuates the glycemic and insulinemic response, hence offering a more healthy option for individuals suffering from obesity, diabetes or cardiovascular diseases.^2^

**Scheme 1:**
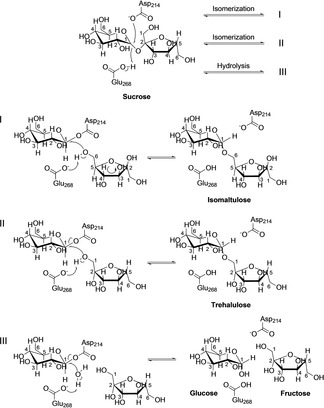
Reaction mechanism for the sucrose isomerase SmuA, which converts sucrose into isomaltulose as well as the by‐products trehalulose, glucose and fructose.

In nature, isomaltulose is found in sugar cane juice as well as honey.^3^ Currently, isomaltulose is produced from sucrose using immobilized bacteria that naturally express an enzyme known as sucrose isomerase (SI; EC 5.4.99.11). This enzyme catalyses the isomerization of sucrose to produce mainly isomaltulose and trehalulose, accompanied by hydrolysis of sucrose to glucose and fructose as by‐products (Scheme 1).^4^

SI enzymes were identified in a range of microbes including *Serratia plymuthica, Pantoea dispersa, Rhizobium sp., Klebsiella planticola* and *Erwinia rhapontici*.^3^, ^5^ Crystal structures of four SIs with distinct product specificities were elucidated: SmuA from *S. plymuthica*,^6^ MutB from *Rhizobium sp*. MX‐45,^7^ *Ks*PalI from *Klebsiella sp*. LX3^8^ and *Er*PalI from *E. rhapontici* NX‐5.^9^ These enzymes share a homologous tertiary structure characterized by a three‐domain organization comprising i) a central (β/α)_8_‐barrel at the N terminus, with an active site architecture typical of the glycoside hydrolase family 13, ii) a loop‐rich subdomain and iii) two antiparallel β‐sheets at the C terminus.6b, 8

Despite similar three‐dimensional structure, these enzymes vary in kinetic efficiency and product ratio depending on the pH and reaction temperature. The highest product specificities, either for isomaltulose or for trehalulose, another alternative sweetener, were reported for the SIs from *P. dispersa* UQ68 J (here dubbed PadU; 91 % isomaltulose and 3 % trehalulose at 30–35 °C)5d and from *Rhizobium sp*. MX‐45 (8 % isomaltulose and 91 % trehalulose at 20 °C), respectively.5b Further SIs described in the literature, including SmuA from *S. plymuthica,* predominantly produce isomaltulose (66–86 %), accompanied by minor amounts of trehalulose (9–21 %) as well as fructose and glucose.

In the α‐glucosidase family 13, residues His118, Asp214, Glu268, His341, and Asp342 (numbering refers to SmuA)6b are highly conserved and appear functionally responsible for the isomerization and/or hydrolysis of the glycosidic linkage. The catalytic triad, comprising Asp214, Asp342 and Glu268, is crucial for the first step in the course of the double displacement reaction mechanism. Asp214 acts as a nucleophile that attacks C1 of the glucose moiety, Asp342 is involved in the stabilization of the transition state, and Glu268 acts as a proton donor/acceptor.^10^ Of note, the side chains of Asp214 and Asp342 also interact via hydrogen bonding with N‐5 and O‐2 of the inhibitor deoxynojirimycin, whereas the proton donor Glu268 only interacts via a water molecule with this ligand in the published crystal structure.6b

After nucleophilic attack by Asp214 on the C1 atom of the glucose moiety in sucrose and formation of a covalent intermediate, there are three possible reaction paths which lead to i) isomaltulose, ii) trehalulose or iii) the hydrolysis product, that is, the monosaccharides glucose and fructose (Scheme 1). In the first isomerization pathway to isomaltulose, the fructose moiety performs a 180° rotation, followed by nucleophilic attack of its 6‐OH group at the C1 atom of the glucose moiety, which results in an α‐1,6‐glycosidic linkage. In the second isomerization pathway to trehalulose, the fructose moiety keeps its position, and nucleophilic attack of its 1‐OH leads to the α‐1,1‐glycosidic bond. In the third pathway, a deprotonated water molecule performs a nucleophilic attack at the C1 atom, resulting in the liberation of glucose and fructose.10b, 11

During the industrial production of solid (crystalline) isomaltulose, the down‐stream process is impeded by the presence of hygroscopic trehalulose. Therefore, the separation of isomaltulose and trehalulose is accomplished by an additional centrifugation. This yield‐decreasing step is essential to obtain a powdery isomaltulose product. Consequently, a product mixture with a high percentage of isomaltulose and minimal trehalulose content is highly desirable.^12^

Disappointingly, previous endeavours of enzyme engineering to reduce trehalulose formation with the aim of industrial application of the aforementioned SIs have resulted in a decreased yield of isomaltulose in conjunction with increased production of trehalulose, glucose and fructose and, generally, severe loss of activity.5c, 9, 13 Main target regions for protein engineering in these studies were the isomerization motif ^298^RLDRD^302^ and the so‐called aromatic clamp residues Phe270/Phe294. Mutations of Arg298 and Asp302 altered the enzyme kinetics and product specificity of *Ks*PalI as well as SmuA towards the production of trehalulose.5c, 13a Mutations at the aromatic clamp, which was reported to play a key role in the catalytic mechanism by controlling substrate entry and product exit, abolished both isomaltulose and trehalulose synthesis activity of *Er*PalI and SmuA.^9^, ^14^ Furthermore, mutations in the first shell around the active centre led to a repurposing of SmuA from an isomaltulose to an isomelezitose synthase.^14^

Thus, the demand for an engineered variant of SmuA with better product specificity towards isomaltulose and high enzyme activity has remained an issue. Here, we present a novel approach to improving the corresponding biocatalytic properties of SmuA by way of semirational protein engineering.

## Results and Discussion

### Development of an efficient expression system for recombinant SmuA and analysis of its quaternary structure

In a first attempt, SmuA was overproduced in the cytoplasm of *Escherichia coli* at the shake flask scale using the expression vector pASK‐IBA5(+), which uses the chemically inducible tetracycline promoter.^15^ After systematic variation of inducer concentration, growth temperature and cultivation time, the highest protein yield of 7.9 mg/L, with a percentage of soluble (folded) protein of 50 %, was achieved after induction of recombinant gene expression with 20 μg/L anhydrotetracycline at 16 °C for 22 h (see the Experimental Section). In an attempt to further increase the percentage of soluble folded protein by protein engineering, several hydrophobic amino acids exposed on the surface of SmuA were replaced by hydrophilic side chains. The substitution of Val465 by Glu boosted the recombinant protein yield to 19.6 mg/L, with an increased proportion of soluble folded protein of 72.5 %. This mutant SmuA(V465E) was chosen for further studies.

**Figure 1:**
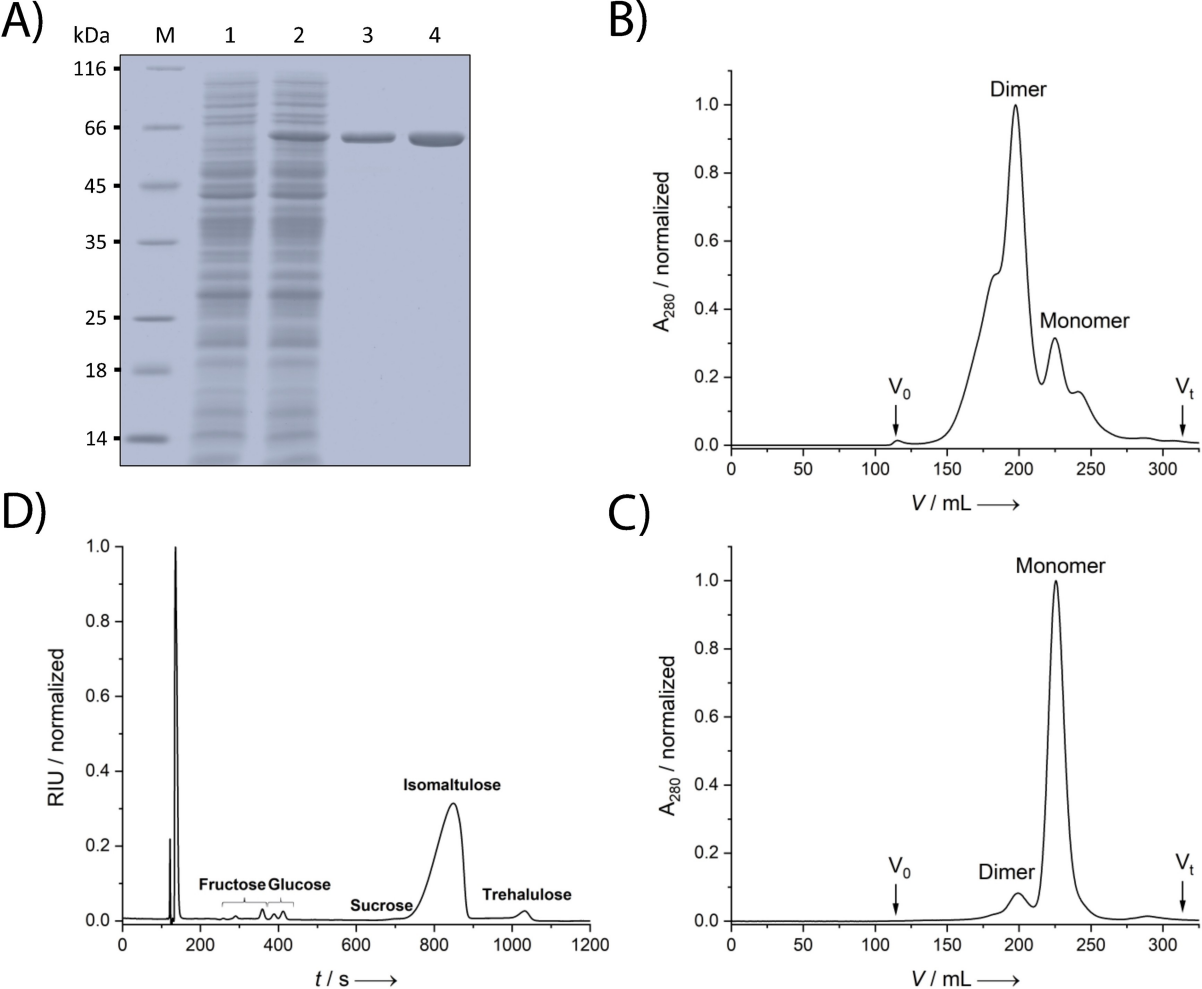
Purification of SmuA(465E) produced in *E. coli* and its enzymatic analysis. A) Overview of the cytoplasmic production and purification of recombinant SmuA(V465E) analysed by SDS‐PAGE. Lanes: M, molecular size marker, 1: total cell lysate of *E. coli* strain BL21 harbouring pASK‐IBA5(+)‐SmuA prior to induction, 2: total cell lysate after 22 h of recombinant gene expression at harvest, 3: pooled elution fractions after SAC purification, 4: final preparation of SmuA(V465E) after SEC. B) SEC profile of SmuA(465E) expressed in the cytoplasm of BL21 after SAC. C) SEC profile of SmuA(465E) expressed in the periplasm in the strain KS272 after SAC purification. D) HPLC analysis as part of the enzyme activity assay of SmuA, revealing the different isomerization and hydrolysis products. Chromatographic conditions: XBridge amide column, detection by using the Smartline refractive index detector 2300. The enzyme activity assay was carried out in 25 mM calcium acetate, adjusted to pH 5.5 with HCl, in the presence of 1.16 M sucrose and 74.4 nM of the recombinant enzyme for 16 h at 20 °C.

In the next step, this enzyme was purified from the total protein extract of *E. coli* by Strep‐Tactin affinity chromatography (SAC) with the help of the Strep‐tag II,^16^ which had been appended to its N terminus. Unexpectedly, the elution profile in the following preparative size exclusion chromatography (SEC) showed two distinct peaks, indicating a dimer and a monomer at a ratio of 3 : 1 (Figure 1). For comparison, the published crystal structure of SmuA (PDB ID: 3GBE)6b suggested a monomeric state while biochemical evidence on the formation of a dimer has not been reported to date. Changes in buffer conditions after purification via SEC, like pH and salt concentration, addition of calcium as well as the use of different buffering agents had no influence on the oligomerization state. Therefore, it appeared that the ratio between monomer and dimer was already determined during protein biosynthesis in the bacterial cytoplasm.

**Figure 2:**
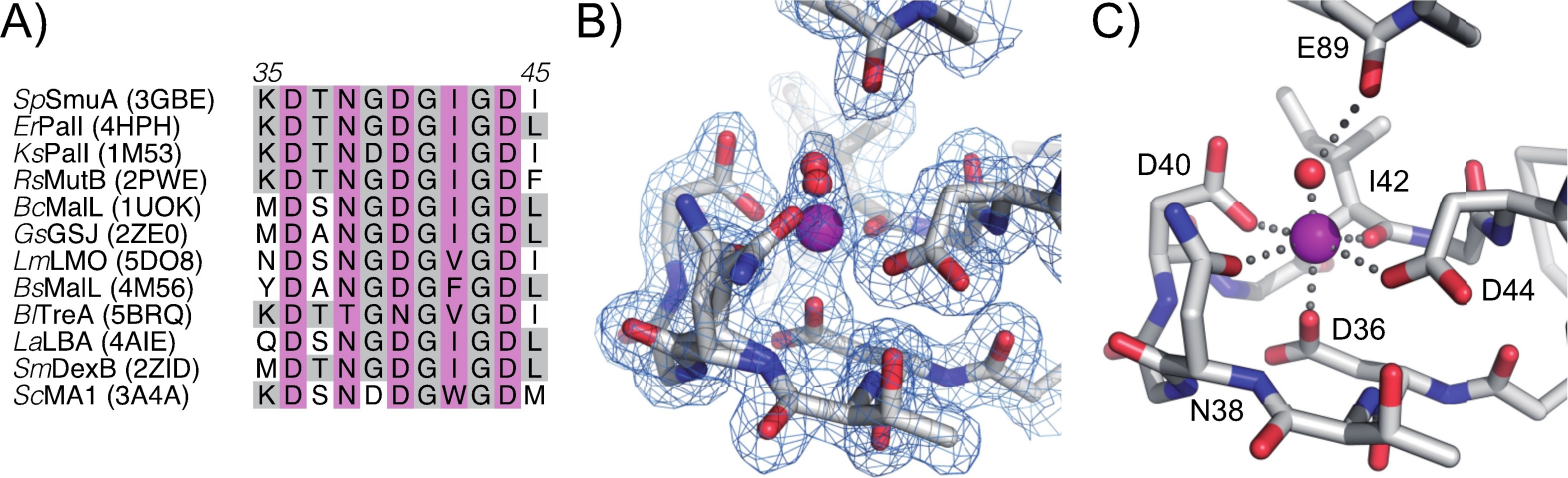
A calcium binding site in SmuA. A) Sequence alignment of the calcium‐binding motifs of SmuA and structurally related enzymes whose coordinate sets are available from the PDB. Metal‐coordinating residues are highlighted in magenta. Residues Asp36, Gly41, Gly43 and Asp44 (cf. panel C) are strictly conserved. B) Refinement of the calcium binding site based on the deposited structure factors (PDB ID: 3GBE). The 2*F*_O_‐*F*_C_ electron density map (blue mesh) is contoured at 1 *σ*, indicating ca. 50 % occupancy for the bound calcium ion as well as two alternative conformations/locations for the calcium coordinating Asn38 side chain and a water molecule, depending on the presence or absence of the calcium ion (cf. the Experimental Section). C) Coordination sphere for a fully occupied calcium binding site, in which Asn38 and the water molecule occupy distinct positions.

Searching the scientific literature for dimer‐forming enzymes homologous to SmuA revealed the α‐glucosidase GSJ from *Geobacillus sp*., which likewise belongs to the glycoside hydrolase family 13.^17^ However, closer inspection of the experimental evidence for the GSJ dimer,^18^ as well as a contact analysis of its crystal structure, suggested that dimer formation is unlikely to be physiologically relevant. Moreover, all other SmuA homologues with known three‐dimensional structure behave as monomers. Notably, a detailed analysis of the primary structure revealed that SmuA shares a calcium‐binding motif (^36^DTNGDGIGD^44^) with its structural homologues, for example MutB13b (Figure 2), which has not been reported so far. When introducing a double substitution of Asp44 by Lys and of Asn38 by Asp in this peptide segment a significantly reduced percentage of soluble folded protein (8 %) was observed, which proves the crucial role of the calcium‐binding site for the efficient folding of SmuA. This notion prompted us to perform a new refinement of the crystal structure of this enzyme using the deposited structure factors (PDB ID: 3GBE). Indeed, the newly calculated electron density map verified the presence of a bound calcium ion (Figure 2B, C) instead of the water molecule that had been placed at the centre of the loop in the published structural model6a (cf. the Experimental Section). Nevertheless, despite this structural rationale, neither addition of calcium acetate to the purified protein nor depletion of calcium ions with EDTA had an effect on the monomer/dimer ratio.

Of note, the uptake of calcium ions into the bacterial cytoplasm is tightly regulated and its natural concentration is very low, with 90 nM.^19^ Consequently, addition of calcium to the culture medium is not expected to influence the folding of the enzyme if directly expressed in the cytoplasm. However, the natural SmuA gene product carries a signal peptide at its N terminus which directs export into the periplasm of *S. plymuthica*.5d Thus, we decided to express SmuA also in the periplasm of *E. coli* strain KS272 using the expression plasmid pASK‐IBA4(+)‐SmuA, which encodes an N‐terminal OmpA signal sequence.^15^ In addition, we supplemented the culture medium with 1 mM calcium acetate to create a calcium‐rich environment during protein expression and folding, anticipating that outer membrane porins should allow free cation diffusion into the periplasmic space. After Strep‐tag II affinity purification from the periplasmic cell fraction, the elution profile of the SEC showed a drastic improvement of the ratio between monomer and dimer to 10 : 1 under these conditions (Figure 1C). Taken together, these findings confirmed the crucial role of calcium during protein folding of SmuA and demonstrated that the monomer is the functional species of this enzyme, which can be readily obtained if appropriate expression conditions are applied. Interestingly, in activity assays (see below) the dimer fraction, isolated by preparative SEC, also turned out to be functional, but with a threefold lower specific activity compared to the monomeric enzyme.

### Semirational mutagenesis strategy and iterative improvement of SmuA product specificity

Generally, the directed evolution of enzymes – and also of binding proteins such as antibody fragments or alternative scaffolds^20^ – relies on the deliberate variation of a protein sequence at a defined level of randomness. Starting from insight into the molecular structure, either single amino acid positions or entire ranges of the amino acid sequence can be mutagenized and resulting libraries subjected to specific screening and/or selection strategies. This concept usually involves an iterative process consisting of i) creating a mutant library, ii) expressing and, optionally, isolating the mutants and iii) screening their enzymatic activities (or ligand affinities) until a satisfactory level of performance is reached.^21^ In the present case, the development of a powerful high throughput screening system based on the direct spectroscopic measurement of either isomaltulose or trehalulose as products of SmuA catalysis was not feasible. To quantify enzyme activity and product distribution we had to establish HPLC analysis on an amide‐functionalized column with a refractive index detector (cf. the Experimental Section), which allowed us to precisely detect even low percentages of trehalulose. Consequently, we needed a strategy that offered an appropriate balance between screening effort via product analysis at the single mutant level and reliable input from rational protein engineering experiments based on the known crystal structure of this enzyme.

With this goal in mind, we were inspired by the board game “battleship”, which comprises repeated rounds between two players in which a shot is fired at the opponent's fleet on a marked paper grid. Each shot directed at a selected position on that grid is either marked as “hit” or “miss”. Upon feedback by the other player, the attacking player notes these results on his own tracking grid in order to gradually build a scheme of the opponent's fleet. In this way a characteristic hit pattern emerges, which is used to plan the next shot until the positions and shapes of all ships have been precisely spotted by one of the players and the game ends. A similar strategy was here employed for the semirational protein engineering of SmuA by replacing amino acid positions in the vicinity of the active site, followed by functional characterization, in an iterative manner. In total, we analysed 55 single point mutations at 30 different amino acid positions (Table 1), eventually yielding enzyme candidates with clearly improved properties.

**Table 1:** Product composition of SmuA and its mutants. The enzyme activity assay was carried out in Ca(OAc)_2_/HCl pH 5.5 containing 1.16 M sucrose for 16 h at 20 °C, followed by product quantification via HPLC analysis. Activity assays for the most promising mutants were carried out as triplicates and the standard deviations are reported. Mutants which were selected for subsequent combination are highlighted in bold letter type.

	Mutant	Yield [%]		
		Isomaltulose	Trehalulose	Monosaccharides
	SmuA(V465E)	93.7±0.02	3.5±0.08	2.8±0.09
Cycle 1	S303P	87.9	8.2	3.9
	D302Y/S303P	94.5	3.6	1.9
	R429K	48.8	4.3	46.9
Cycle 2	A217V	82.8	1.7	15.5
	T427I	85.9	5.4	8.7
	T427S	92.0±0.10	3.8±0.07	4.2±0.03
	T427V	87.0	4.5	8.5
	D430E	87.1	3.5	9.4
	D430N	84.4±0.22	3.4±0.12	12.2±0.21
Cycle 3	D430A	81.1	3.9	14.9
	D430L	79.1	3.7	17.2
	D430Q	81.9	3.7	14.4
	D430S	86.3±0.09	3.1±0.09	10.6±0.17
	D430T	86.9±0.10	2.9±0.11	10.1±0.20
Cycle 4	F236Y	92.9±0.15	3.8±0.15	3.3±0.01
	I269A	90.2	5.3	4.5
	**I269V**	**94.6±0.12**	**3.1±0.09**	**2.3±0.04**
	**S277T**	**91.7±0.16**	**2.5±0.08**	**5.8±0.10**
	**D311P**	**93.2±0.08**	**3.3±0.04**	**3.5±0.04**
	D399N	93.0±0.02	3.7±0.05	3.3±0.04
	I400A	77.8	5.4	16.8
	I400V	92.5	3.9	3.6
	S428A	91.1	4.4	4.5
	S428T	90.9	3.9	5.1
Cycle 5	Y78W	43.9	5.9	50.2
	A179V	90.4	3.8	5.8
	I297G	72.6	4.1	23.3
	L299G	93.1	3.9	3.0
	H341F	81.0	1.1	17.9
	**D398G**	**94.7±0.18**	**2.7±0.18**	**2.6±0.04**
	D398P	93.0	3.7	3.3
	D399E	72.2	4.8	23.0
	D399S	0.42	0.42	99.2
	I400Y	80.4	3.0	16.6
	E401Q	6.1±0.18	7.8±0.27	86.1±0.23
	**F453Y**	94.6±0.11	2.5±0.15	2.9±0.06
Cycle 6	P67S	74.8±0.61	1.3±0.23	23.9±0.75
	**F270Y**	**95.2±0.09**	**3.2±0.03**	**1.6±0.07**
	I269L	89.1±0.33	8.0±0.21	2.9±0.13
	S277M	93.1±0.05	4.4±0.06	2.5±0.05
	**S277Q**	**92.8±0.14**	**2.4±0.08**	**4.8±0.07**
	E401D	77.5±0.92	2.3±0.33	20.2±0.94
	N431Q	84.4±0.53	13.8±0.50	1.8±0.04
	N431S	87.3±0.27	3.3±0.09	9.4±0.20
	F453L	93.0±0.23	3.7±0.18	3.3±0.07
	F453W	91.0±0.27	2.8±0.21	6.2±0.10
Cycle 7	K180G	94.0±0.07	3.6±0.08	2.4±0.04
	K180V	93.7±0.11	4.0±0.05	2.3±0.07
	**Q181P**	**94.1±0.05**	**2.0±0.05**	**3.8±0.03**
	F213L	93.5±0.13	3.6±0.13	2.9±0.02
	**V216A**	**94.1±0.17**	**2.0±0.09**	**3.9±0.10**
	**Y219L**	**94.7±0.04**	**1.9±0.02**	**3.4±0.04**
	F294Y	86.1±0.08	0.9±0.11	13.0±0.06
	**T369F**	**94.8±0.09**	**2.3±0.06**	**2.9±0.11**
	T369W	93.8±0.08	3.9±0.07	2.3±0.01
	**L426V**	**94.8±0.08**	**2.8 ±0.12**	**2.4±0.07**
	**L426Y**	**94.7±0.08**	**2.6±0.08**	**2.7±0.04**

The starting variant SmuA(V465E) described above showed a product spectrum comprising 93.7 % isomaltulose, 3.5 % trehalulose and 2.8 % monosaccharides, with a ratio of isomaltulose to trehalulose of 26.8 : 1. Known as the best isomaltulose producing enzyme so far,^22^ the sucrose isomerase PadU from *P. dispersa* showed in the same assay a product spectrum of 96.3 % isomaltulose, 1.2 % trehalulose and 2.5 % monosaccharides, thus setting the benchmark with an isomaltulose/trehalulose ratio of 80.9 : 1. However, its inferior catalytic activity compared with SmuA (see below) as well as the lack of a crystal structure provided the rationale for focusing at the latter enzyme in this protein engineering endeavour.

Our initial three cycles of SmuA engineering focused on the first shell of amino acid residues around the active centre. Mutations at the positions Ala217, Thr427, Lys429 and Asp430 (Table 1) led to an undesired increase in trehalulose production together with a severe drop in catalytic activity. This rendered the evolutionarily conserved immediate vicinity of the active site5c, 6b inappropriate for further mutagenesis attempts. Consequently, our next efforts aimed at the second or third shell of amino acid positions. Apart from a couple of further misses, at Phe236, Ile400 and Ser428 (cf. Table 1), we succeeded during the 4th mutagenesis cycle with our first hits, that is, the amino acid exchanges I269V, S277T and D311P. Thus, each analysed mutant, no matter whether hit or miss, increased our understanding of enzyme regions that play a role in the isomerization of sucrose to isomaltulose, and corresponding findings served in the decision making for the next mutant cycle. Thereby, the chances of identifying mutants with beneficial characteristics gradually increased during each round, culminating in the most successful 7th cycle with six hits out of eleven mutations that were tested.

**Figure 3:**
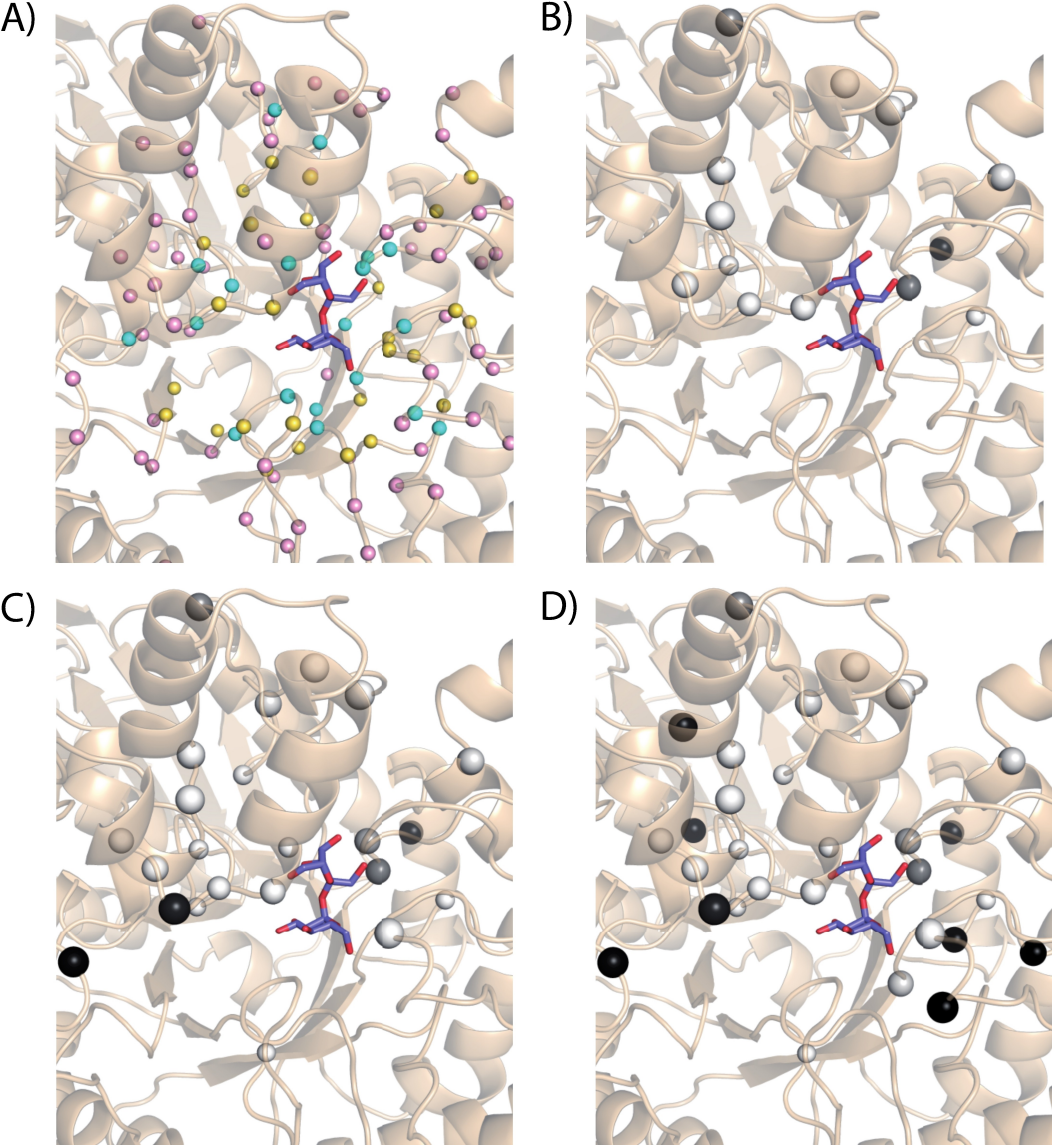
Structural overview of the mutational hit pattern at different stages of the semirational engineering of SmuA(V465E) based on the crystal structure of the wild type enzyme (cf. Fig. 2). To create a model of sucrose bound at the active center of SmuA, crystal structures of SmuA (PDB ID: 3GBE) with the bound inhibitor deoxynojirimycin and of MutB (PDB ID: 2PWE) with bound sucrose were superimposed via the Cα positions. Subsequent removal of deoxynojirimycin and the MutB polypeptide chain led to the depicted model. (A) Overview of the three shells of residues in SmuA around the sucrose substrate. Residues with atoms up to 4 Å from sucrose were assigned as the first shell (cyan), those in a distance of 4–8 Å as the second shell (yellow) and those at 8–12 Å distance as the third shell (pink), considering that 12 Å was the farthest distance of any mutated position. Although located at a distance >4 Å we assigned both Arg^298^ and Arg^301^ (part of the “isomerization motif”) to the first shell as their side chains directly point at the fructose moiety. (B) Stage I of the battleship mutagenesis campaign including cycles 1, 2 and 3; (C) Stage II including cycles 4 and 5; (D) Stage III including cycles 6 and 7. Each mutated amino acid residue is displayed as a sphere around the Cα position to illustrate the effect of the corresponding substitution (i) by the sphere size (increasing catalytic activity) and (ii) by the gray shade (ratio isomaltulose/trehalulose). Mutants with an isomaltulose/trehalulose product ratio of ≤26.8 : 1 are represented by a white sphere, whereas increased product specificity towards isomaltulose is shown in darker shades. This illustration was prepared using PyMOL software.^23^

In the course of this iterative screen (Table 1), in total 13 hits with decreased formation of the undesired by‐product trehalulose were identified, while maintaining overall high catalytic activity. The activity and product specificity values that were individually determined for each mutant enzyme led to an evolving pattern, which permitted graphical representation with positive hits mapped on the three‐dimensional enzyme structure as spheres with increasing sizes and shades of grey (Figure 3). The best single mutation, Y219L, doubled the ratio of isomaltulose to trehalulose from 26.8 : 1 to 52.3 : 1. Consequently, this mutation significantly decreased the total production of trehalulose from 3.5 to 1.8 %. Other mutations showed a reduced trehalulose production in the range of 2.0–3.3 %, yet often combined with an increased percentage of the hydrolysis by‐products (glucose and fructose). In this regard, the mutation F270Y is worth mentioning due to its low production of only 1.6 % monosaccharides along with a reduced production of trehalulose. This is in line with the previously postulated role of the two aromatic clamp residues Phe270 and Phe294 in controlling the entrance of the substrate to the active site as well as exit of products.5c, 6b, 13b In earlier reports, however, mutation of either one these Phe residues to Ala abolished the isomerization activity of SmuA, just retaining the hydrolytic activity.^9^, ^14^

**Figure 4:**
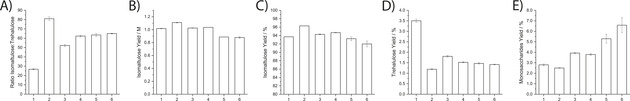
Enzyme activity assays of SmuA and its mutants in comparison with PadU. 1) SmuA(V465E), 2) PadU, 3) SmuA(Y219L/V465E), 4) SmuA(Y219L/D398G/V465E), 5) SmuA(Q181P/D398G/F453Y/V465E), 6) SmuA(Y219L/T369F/D398G/F453Y/V465E). A) Ratio of isomaltulose to trehalulose; B) Isomaltulose yield; percentage yield of C) isomaltulose, D) trehalulose and E) the monosaccharides. The enzyme activity assay was carried out in Ca(OAc)_2_/HCl pH 5.5 containing 1.16 M sucrose for 16 h at 20 °C, followed by product quantification by HPLC analysis.

In the next stage, we combined the most promising single mutations in SmuA, which led to an successive improvement of product specificity (Table 2). In fact, the best variant, SmuA(Y219L/T369F/D398G/F453Y/V465E), produced only 1.4 % trehalulose and showed an isomaltulose/trehalulose ratio of 65.0 : 1. Nevertheless, the progressive reduction in trehalulose yield also correlated with an increased production of glucose and fructose up to 6.6 % (Figure 4).

**Table 2:** Product composition, ratio isomaltulose/trehalulose and isomaltulose yield for SmuA and its mutants in comparison with PadU.

SmuA	Yield [%]			Ratio	Isomaltulose
mutant	Isomaltulose	Trehalulose	Monosaccharides	Isomaltulose/ Trehalulose	Yield [M]
SmuA (V465E)	93.7±0.02	3.5±0.08	2.8±0.09	26.8±0.59	1.0±0.01
PadU	96.3±0.01	1.2±0.03	2.5±0.03	80.9±1.98	1.1±0.01
Q181P	94.1±0.05	2.0±0.05	3.8±0.03	46.1±1.17	1.0±0.01
V216A	94.1±0.17	2.0±0.09	3.9±0.10	48.4±2.19	0.9±0.01
Y219L	94.3±0.12	1.8±0.04	3.9±0.08	52.3±1.2	1.0±0.01
I269V	94.6±0.12	3.1±0.09	2.3±0.04	30.9±0.92	1.0±0.02
F270Y	95.2±0.09	3.2±0.03	1.6±0.07	30.0±0.34	0.9±0.01
S277Q	92.8±0.14	2.4±0.08	4.8±0.07	38.3±1.41	1.0±0.01
S277T	91.7±0.16	2.5±0.08	5.8±0.10	36.9±1.26	0.9±0.01
D311P	93.2±0.08	3.3±0.04	3.5±0.04	28.4±0.35	1.0±0.01
T369F	94.8±0.09	2.3±0.06	2.9±0.11	41.2±1.03	1.0±0.01
D398G	94.7±0.18	2.7±0.18	2.6±0.04	35.1±2.47	1.0±0.02
L426V	94.8±0.08	2.8±0.12	2.4±0.07	34.4±1.54	1.1±0.02
L426Y	94.7±0.08	2.6±0.08	2.7±0.04	37.0±1.11	1.0±0.01
F453Y	94.6±0.11	2.5±0.15	2.9±0.06	38.5±2.29	1.0±0.01
Y219L/D398G	94.7±0.06	1.5±0.02	3.8±0.08	62.4±0.96	1.0±0.01
Q181P/D398G/F453Y	93.2±0.44	1.5±0.03	5.3±0.41	63.5±1.62	0.9±0.02
Y219L/T369F/D398G/F453Y	92.0±0.69	1.4±0.02	6.6±0.71	65.0±0.38	0.9±0.01

### Detailed kinetic and stability analysis of the engineered SmuA versions

**Figure 5:**
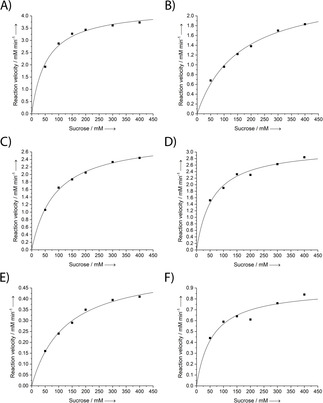
Michaelis‐Menten kinetics for the production of isomaltulose by SmuA and its mutants in comparison with PadU. A) SmuA(V465E); B) PadU; C) SmuA(Y219L/V465E); D) SmuA(Y219L/D398G/V465E); E) SmuA (Q181P/D398G/F453Y/V465E); F) SmuA(Y219L/T369F/D398G/F453Y/V465E). For the resulting Michaelis constant (*K*_M_), the turnover number (*k*_cat_) and the catalytic efficiency (*k*_cat_/*K*_M_) of each enzyme version, see Table 3.

To allow a more precise assessment of the practical value of the improved SmuA mutants, we determined their kinetic parameters and compared these with the ones obtained for the recombinant PadU (Figure 5, Table 3). In our assay, SmuA(V465E) showed a low *K*_M_ value of 55.3 mM at 20 °C, which is similar to the previously published *K*_M_=65 mM at 30 °C for the native enzyme.^24^ Notably, PadU exhibited a threefold higher *K*_M_ value of 157 mM and a considerably lower activity (*k*_cat_=567 s^−1^, *k*_cat_/*K*_M_=3.6 mM^−1^ s^−1^) under the same experimental conditions – versus a reported *K*_M_=40 mM in 0.1 M citrate/phosphate buffer pH 6.0 at 30 °C.5d

**Table 3:** Kinetic parameters for the production of isomaltulose by SmuA and its mutants in comparison with PadU.

SmuA mutant	*K*_M_	*k*_cat_	*k*_cat_/*K*_M_
	[mM]	[s^−1^]	[mM^−1^ s^−1^]
SmuA (V465E)	55.3±6.80	965±30.1	17.5±1.31
PadU	157±17.8	567±27.1	3.6±0.29
Y219L	86.6±5.47	665±13.4	7.7±0.31
Y219L/D398G	60.2±9.71	708±30.3	11.8±1.20
Q181P/D398G/F453Y	124±12.6	122±4.70	0.99±0.07
Y219L/T369F/D398G/F453Y	58.2±17.4	201±15.7	3.5±0.66

In our measurements we observed significant changes in the kinetic parameters for the various mutants of SmuA. Compared to the reference enzyme, SmuA(V465E), all variants showed moderately increased *K*_M_ values towards sucrose in the range of 58.2–86.6 mM and, accordingly, slightly decreased catalytic efficiencies (Table 3). Among those, the triple mutant SmuA(Y219L/D398G/V465E) showed the highest *k*_cat_/*K*_M_ ratio of 11.8 mM^−1^ s^−1^ – that is three‐fold the efficiency of PadU.

**Figure 6:**
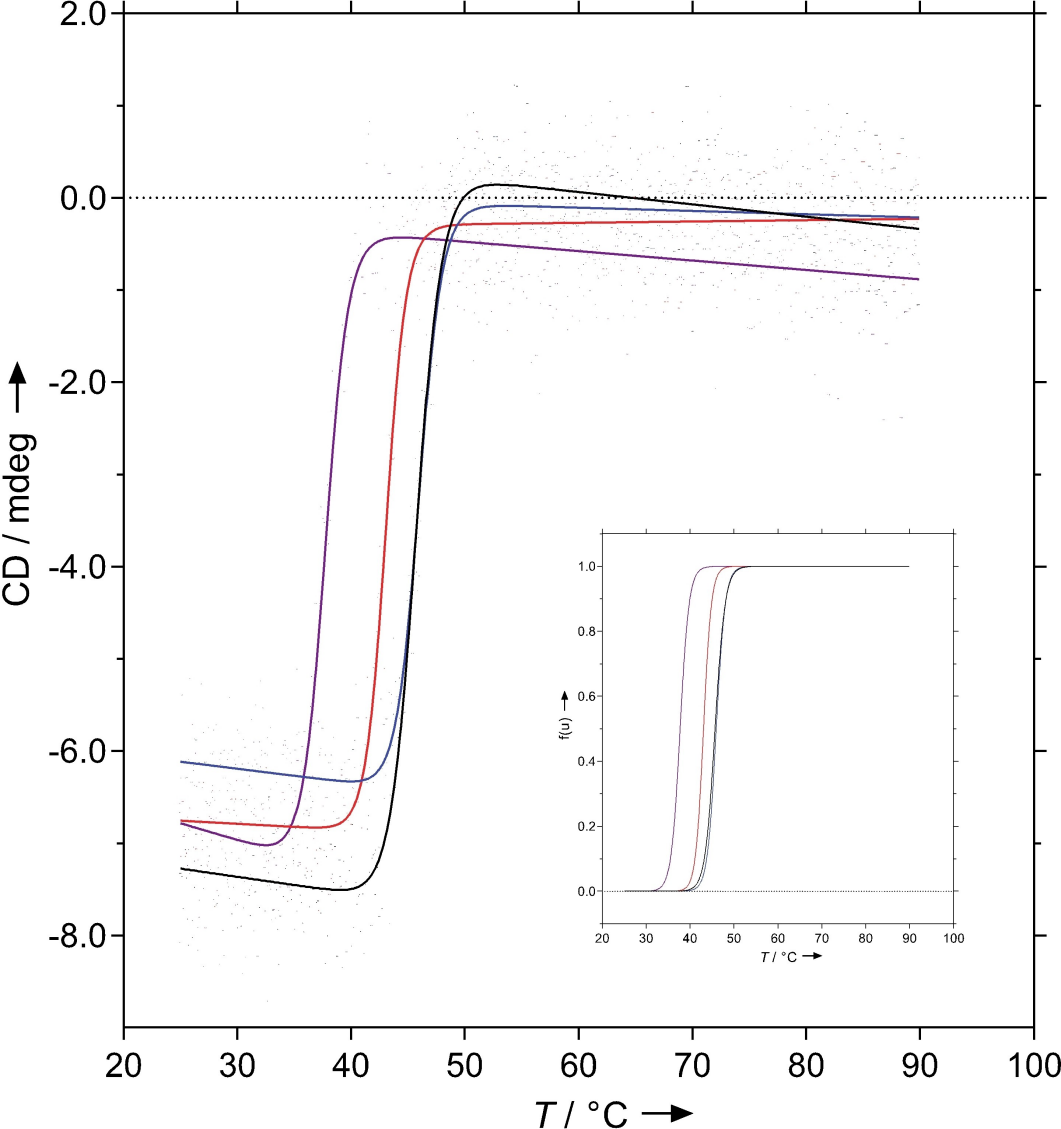
Melting curves of the most promising mutants with the wild‐type enzyme: SmuA, black; SmuA(V465E), blue; SmuA(Y219L/D398G/V465E), red; SmuA(Y219L/T369F/D398G/F453Y/V465E), purple. The CD signal is plotted versus the temperature. The inset depicts normalized plots of the fraction unfolded f(u) versus temperature (using the same colouring scheme).

To assess the influence of mutations on the folding stability of the functionally optimized SmuA mutants we performed thermal unfolding studies for the reference enzyme and the most important variants by monitoring the circular dichroism (CD) signal at 210 nm (Figure 6). As result, recombinant wild‐type SmuA and SmuA(V465E) showed almost identical *T*_m_ values of 45.7 and 46 °C, respectively. Introduction of the additional mutations Y219L/D398G resulted in a slightly lower melting temperature of 43.0 °C for the triple mutant, whereas the *T*_m_ value dropped to 37.7 °C in case of the four substitutions Y219L/T369F/D398G/F453Y. The cooperativity of unfolding (▵*H*_m_) was in the range of 750–927 kJ/mol for all measurements.

Taken together, with a catalytic efficiency of *k*_cat_/*K*_M_=11.8 mM^−1^ s^−1^ for sucrose isomerization and a very low trehalulose yield of 1.5 % as well as efficient production in *E. coli* and a *T*_m_ value well above the assay/working temperature, the SmuA triple mutant Y219L/D398G/V465E appeared particular promising for industrial use in the biocatalytic preparation of isomaltulose with improved product quality.

## Conclusions

In this study, we describe a novel strategy for the semirational protein engineering of an enzyme inspired by the battleship board game. This game's principle was transferred to the directed evolution of a biocatalyst under somewhat challenging conditions as SmuA represents a sucrose isomerase with already fairly high product specificity. Since a simple chromogenic readout was not feasible, precise analysis of the product spectrum using laborious HPLC methodology was required in order to reliably identify mutants that showed minute improvements, in particular with regard to less formation of the detrimental by‐product trehalulose.

As result, we succeeded in engineering the highly active sucrose isomerase SmuA for even better product specificity. The trehalulose by‐product percentage was iteratively decreased from 3.5 % to as low as 1.4 % in case of the “quadruple” mutant SmuA(Y219L/T369F/D398G/F453Y/V465E) which additionally carried one mutated surface residue for enhanced solubility. However, among the set of combination mutants with superior properties the triple mutant SmuA(Y219L/D398G/V465E) offered the best compromise and a promising alternative to the established sucrose isomerase PadU due to its low trehalulose production of 1.5 % in combination with a high catalytic efficiency (*k*_cat_/*K*_M_=11.8 mM^−1^ s^−1^). This mutant SmuA enzyme bears potential for applications in biotechnology and food industry to improve the current process of isomaltulose production.

## Experimental Section

**Cloning and site‐directed mutagenesis of the sucrose isomerase SmuA**: The synthetic *smuA* structural gene, whose encoded amino acid sequence corresponds to UniProt ID: D0VX20 (containing one additionally inserted Asp residue at position 380), was amplified from pET28a‐SmuA (provided by Evonik Industries) using Q5 High Fidelity DNA Polymerase with the primers 5′‐GCC ACC CAG CAG CCG CTG CTG‐3′ and 5′‐CGA TCA AAG CTT ACT GGT TCA GTT TGT AAA CAC CGG ACT GCC‐3′, which carry recognition sites (underlined) for EheI (only in part) and HindIII, respectively. After restriction digest with HindIII the PCR product was ligated with the backbones of the vectors pASK‐IBA4(+) and pASK‐IBA5(+),^15^, ^16^ which had been cut with the two corresponding restriction enzymes. While both vectors encode an N‐terminal Strep‐tag II, pASK‐IBA5(+)‐SmuA was used for cytoplasmic expression whereas pASK‐IBA4(+)‐SmuA also carries an N‐terminal OmpA signal sequence and was used for periplasmic expression.

SmuA variants, including the deletion of the extraneous Asp residue, were generated by site‐directed mutagenesis using the QuikChange mutagenesis method (Agilent, Waldbronn, Germany) with suitable primers. Resulting expression plasmids were confirmed by restriction analysis with KasI and HindIII as well as automated DNA sequencing (Eurofins Genomics, Ebersberg, Germany).

**Cytoplasmic expression of SmuA and its mutants in** ***E. coli*** **and enzyme purification**: *E. coli* BL21^25^ transformed with pASK‐IBA5(+)‐SmuA was grown at 37 °C in 2 L LB medium^26^ supplemented with 100 μg/mL ampicillin in a 5 L shake flask to OD_550_=0.5. Recombinant gene expression was induced upon addition of 0.2 μg/mL anhydrotetracycline (Acros Organics, Geel, Belgium), and cultivation was continued for 16 h at 16 °C. Bacteria were harvested by centrifugation and resuspended in 20 mL 100 mM Tris/HCl pH 8.0 and 1 mM EDTA. After cell disruption using a French pressure cell (SLM Aminco, Urbana, IL), the raw extract was centrifuged at 15,000 rpm for 30 min at 4 °C.

The soluble whole‐cell protein extract was sterile‐filtrated and applied onto a Strep‐Tactin Superflow column (IBA, Göttingen, Germany) with 2 mL bed volume, which had been pre‐equilibrated with SA buffer (100 mM Tris/HCl pH 8.0, 150 mM NaCl, 1 mM EDTA). After washing with SA buffer, the bound protein was eluted with 2.5 mM D‐desthiobiotin (IBA) in the same buffer. The elution fractions were concentrated by ultrafiltration (0.22 μm; Amicon/Merck Millipore, Cork, Ireland) and purified via SEC on a Superdex 200 10/300 GL column (GE Healthcare, Munich, Germany) using 20 mM Tris/HCl pH 8.0, 150 mM NaCl as running buffer. Appropriate elution fractions were collected and tested for purity via SDS‐PAGE and staining with Coomassie brilliant blue R‐250. The precise molecular mass of the purified proteins was determined by electrospray ionization mass spectrometry (ESI‐MS) on a maXis instrument (Bruker, Hamburg, Germany).

**Periplasmic expression of SmuA and its mutants in** ***E. coli*** **and enzyme purification**: *E. coli* KS272^27^ transformed with pASK‐IBA4(+)‐SmuA was grown at 37 °C in 2 L LB medium^26^ supplemented with 1 mM calcium acetate (Sigma‐Aldrich) and 100 μg/mL ampicillin in a 5 L shake flask to OD_550_=0.5. Recombinant gene expression was induced by addition of 0.2 μg/mL anhydrotetracycline, and cultivation was continued for 16 h at 16 °C. Bacteria were harvested by centrifugation and resuspended in 20 mL 100 mM Tris/HCl pH 8.0, 0.5 M sucrose, 1 mM EDTA. After incubation on ice for 30 min, the suspension was centrifuged at 5,000 rpm for 10 min at 4 °C to sediment the spheroplasts and the supernatant was fully clarified by a second centrifugation at 15,000 rpm for 15 min at 4 °C. The resulting periplasmic protein extract was dialysed against SA buffer over night at 4 °C and sterile‐filtrated. The periplasmic protein solution was applied onto a Strep‐Tactin Superflow column and purified as above, followed by SEC and protein analytics.

**Production of PadU in** ***E. coli***: The *padU* coding region, whose amino acid sequence corresponds to the UniProt ID: Q6XNK6, was amplified from pMA‐T_PadU (synthetic gene provided by GeneArt, Regensburg, Germany) using Q5 High Fidelity DNA Polymerase with the primers 5′‐CCA CTC CCT ATC AGT GAT‐3′ and 5′‐CGC AGT AGC GGT AAA CG‐3′. After restriction digest with both KasI and HindIII, whose recognition sites flank the coding region, the PCR product was ligated with the backbone of pASK‐IBA4(+) as described for SmuA above. Subsequent expression and purification of PadU were carried out accordingly.

**Enzyme assay and kinetic analysis of SmuA and its mutants**: Enzyme activity was determined in 25 mM calcium acetate, adjusted to pH 5.5 with HCl, in the presence of 1.16 M sucrose (Sigma‐Aldrich) as substrate by applying 5 μg/mL (74.7 nM) of the purified enzyme for 16 h at 20 °C. After that, the enzymatic product composition was analysed on a 1200 HPLC instrument (Agilent Technologies, Santa Clara, CA) equipped with the Smartline refractive index detector 2300 (Knauer, Berlin, Germany). A sample solution of 20 μL was applied onto an XBridge amide column (particle size: 5.0 μm, inner diameter: 4.6, length: 250 mm; Waters, Milford, MA) at a flow rate of 2 mL/min using isocratic elution with 57.8 % acetonitrile, 26.1 % acetone and 16.1 % H_2_O. The chromatogram was analysed with Origin software (OriginLab, Northampton, MA) by calculating the peak areas. In order to quantify each product, calibration measurements were performed with pure samples of sucrose, isomaltulose, glucose and fructose at concentrations from 10 to 400 mM.

The Michaelis constant (*K*_M_), the turnover number (*k*_cat_) and the catalytic efficiency (*k*_cat_/*K*_M_) for isomaltulose were determined from measurements at sucrose substrate concentrations of 50, 100, 150, 200, 300 and 400 mM, each in a total reaction volume of 2 mL. 500 μL samples were collected after 10, 20, 30 and 40 min, and the enzyme reaction was immediately stopped by heating the sample to 95 °C for 10 min. To determine the initial velocities, the isomaltulose yield was analysed as described above and plotted against the reaction time points of 10–40 min. The slope of the resulting linear regression line was used as the initial velocity value.

**Structural refinement of SmuA from a deposited X‐ray diffraction data set**: Inspection of the SmuA crystal structure (PDB ID: 3GBE)6b revealed a suspicious water position embraced by the loop segment 36–44 with unusually short hydrogen bond distances and an implausible octahedral coordination sphere, as it otherwise is commonly observed for alkali or alkaline earth metal ions. Comparison of SmuA with its structural homologues strongly suggested the presence of a calcium ion at this site. Manual rebuilding and refinement of SmuA using the deposited structure factors were carried out with Coot^28^ and Refmac5,^29^ respectively. Refinement of the calcium ion and its coordination sphere indicated that the binding site was occupied by 50 %. Accordingly, two of the calcium ligands, Asn38 and a water molecule, adopt alternative conformations/positions, depending on the presence of the calcium ion (see text). It appears that a combination of the incomplete occupancy of the calcium binding site as well as the alternative conformations of two of its ligands may have led to the improper modelling of a water molecule in the published crystal structure. Of note, the incomplete occupancy of the calcium site was likely caused by the crystallization buffer, which contained 200 mM lithium citrate (an anion that strongly complexes calcium), whereas the protein solution was supplemented with only 10 mM calcium acetate.6a

**Circular dichroism measurements**: Protein secondary structure was analysed using a J‐810 spectropolarimeter (Jasco, Pfungstadt, Germany) equipped with a 1 mm path length quartz cuvette (106‐QS; Hellma, Müllheim, Germany). For thermal unfolding, solutions of SmuA and its variants (1.5 μM) in 50 mM K_2_SO_4_, 20 mM KP_i_ pH 7.5 were applied in the thermostated cuvette, which was sealed with a teflon lid. The sample was heated from 25 °C to 90 °C at a constant temperature gradient of 60 K/h. Data were collected each 0.1 K step at a wavelength of 210 nm, where maximal spectral change upon unfolding was observed. Data were fitted by non‐linear least‐squares regression using Prism 6 software (GraphPad) and an equation for a two‐state model of the unfolding transition as previously described.^30^ Using the parameters from the corresponding curve fit, the normalized unfolded fraction, f(u), was plotted as a function of temperature.

## Conflict of interest

The authors declare no conflict of interest.
